# Generation of Large Numbers of Antigen-Expressing Human Dendritic Cells Using CD14-ML Technology

**DOI:** 10.1371/journal.pone.0152384

**Published:** 2016-04-06

**Authors:** Yuya Imamura, Miwa Haruta, Yusuke Tomita, Keiko Matsumura, Tokunori Ikeda, Akira Yuno, Masatoshi Hirayama, Hideki Nakayama, Hiroshi Mizuta, Yasuharu Nishimura, Satoru Senju

**Affiliations:** 1 Department of Immunogenetics, Graduate School of Medical Sciences, Kumamoto University, Kumamoto, Japan; 2 Department of Orthopaedic Surgery, Faculty of Life Sciences, Kumamoto University, Kumamoto, Japan; 3 Department of Respiratory Medicine, Faculty of Life Sciences, Kumamoto University, Kumamoto, Japan; 4 Department of Neurology, Faculty of Life Sciences, Kumamoto University, Kumamoto, Japan; 5 Department of Oral and Maxillofacal Surgery, Faculty of Life Sciences, Kumamoto University, Kumamoto, Japan; Baylor College of Medicine, UNITED STATES

## Abstract

We previously reported a method to expand human monocytes through lentivirus-mediated introduction of cMYC and BMI1, and we named the monocyte-derived proliferating cells, CD14-ML. CD14-ML differentiated into functional DC (CD14-ML-DC) upon addition of IL-4, resulting in the generation of a large number of DC. One drawback of this method was the extensive donor-dependent variation in proliferation efficiency. In the current study, we found that introduction of BCL2 or LYL1 along with cMYC and BMI1 was beneficial. Using the improved method, we obtained CD14-ML from all samples, regardless of whether the donors were healthy individuals or cancer patients. *In vitro* stimulation of peripheral blood T cells with CD14-ML-DC that were loaded with cancer antigen-derived peptides led to the establishment of CD4^+^ and CD8^+^ T cell lines that recognized the peptides. Since CD14-ML was propagated for more than 1 month, we could readily conduct genetic modification experiments. To generate CD14-ML-DC that expressed antigenic proteins, we introduced lentiviral antigen-expression vectors and subjected the cells to 2 weeks of culture for drug-selection and expansion. The resulting antigen-expressing CD14-ML-DC successfully induced CD8^+^ T cell lines that were reactive to CMVpp65 or MART1/MelanA, suggesting an application in vaccination therapy. Thus, this improved method enables the generation of a sufficient number of DC for vaccination therapy from a small amount of peripheral blood from cancer patients. Information on T cell epitopes is not necessary in vaccination with cancer antigen-expressing CD14-ML-DC; therefore, all patients, irrespective of HLA type, will benefit from anti-cancer therapy based on this technology.

## Introduction

Vaccination therapies that use antigenic peptides, for example, those emulsified in adjuvant or loaded onto dendritic cells (DC), have been broadly used to treat cancer. During the last two decades, considerable effort has been devoted to identifying cancer antigen-derived CTL epitopes that are restricted to the common alleles of HLA class I, such as HLA-A*02:01 [1–4]. As a result, a vast amount of information has been accumulated on epitopes that are presented by major alleles of HLA class I [5–8]. On the other hand, relatively few epitopes have been identified for low-frequency HLA alleles. Thus, cancer patients who are negative for common types of HLA class I are excluded from most of the currently conducted vaccination therapies. Although HLA-A*02:01 is the most common class I allele worldwide, gene frequency of HLA-A*02:01 is at most 30% in most ethnic groups. Thus, a considerable number of patients cannot benefit from current vaccination therapies [1–4]. In addition, HLA-B-restricted epitopes have hardly been identified, probably due to the absence of particularly dominant alleles in the HLA-B locus. However, there should be many useful HLA-B-restricted epitopes, including already known cancer antigens. If HLA-B-restricted CTLs could also be stimulated, the efficacy of anti-cancer vaccination therapies would be improved substantially.

As a possible means to overcome the restrictions associated with synthetic peptide-based vaccination therapies, gene-based vaccinations, such as plasmid DNA vaccinations or vaccinations using recombinant viruses, may be considered [9, 10]. However, plasmid-based DNA vaccines are not efficient enough to induce anti-cancer immunity [9]. As for therapies using recombinant viruses, the potential risk caused by the administration of infectious virus into patients may be problematic. Vaccination with genetically modified DC expressing cancer antigens may be more efficient and safer [11–13].

DC that are used for anti-cancer therapy are usually generated by *in vitro* differentiation of monocytes in peripheral blood samples, because DC exist in very few numbers in human blood [14, 15]. Genetic modification of monocytes using viral vectors has been reported as a means to generate cancer-antigen-expressing DC [11–13]. Nevertheless, monocytes cannot be propagated, and the selection and expansion of transgenic cells is not feasible. Methods to propagate DC or the precursor monocytes are desirable for a more efficient generation of antigen-expressing DC.

We previously found that lentivirus-mediated transduction of cMYC along with BMI1 induced proliferation of CD14^+^ monocytes [16]. This observation led to the first established method for the amplification of human monocytes, and we named the monocyte-derived proliferating cells CD14-ML. The expanded CD14-ML differentiated into functional DC (CD14-ML-DC) upon the addition of IL-4 to the culture. One drawback to this method was the donor-dependent variation in proliferation induction, and the amplification of CD14^+^ monocytes was unsuccessful in 3 out of 12 blood donors in the previous study [16]. In the current study, we found a way to improve efficiency. In addition, we established a procedure to generate a large number of genetically modified DC expressing antigenic proteins. This method exploits the proliferating capability of CD14-ML. The capacity of CD14-ML-DC to induce vigorous T cell proliferation and cancer antigen-specific T cells demonstrated in this study indicates a potential value in vaccination therapy. Anti-cancer vaccination without information of T cell epitopes will become feasible, based on genetically modified CD14-ML-DC expressing antigenic proteins.

## Materials and Methods

### Cell samples and donors

This study was conducted with the approval of the institutional ethics review board of Kumamoto University, Graduate School of Medical Sciences (Approval numbers: 118 and 499). PBMC samples were collected from healthy donors after obtaining written informed consent. CD14^+^ monocytes of healthy donors purchased from Takara Bio (Otsu, Shiga, Japan) were also used. Blood samples were also collected from 2 head-and-neck cancer (HNC) patients enrolled in a peptide vaccine trial, after obtaining written informed consent. The phase I/II clinical trial of cancer immunotherapy using three HLA-A24-binding short peptides, CDCA1_56–64_, IMP-3_508–516_ and LY6K_177–186_, were approved by the Institutional Review Board of Kumamoto University, Kumamoto, Japan (Approval number: 841), and registered in University Hospital Medical Information Network Clinical Trials Registry (UMIN-CTR; UMIN000008379). UMIN-CTR is approved by ICMJE (International Committee of Medical Journal Editors). In total, monocytes derived from 10 healthy donors and 2 cancer patients were used (Table 1).

**Table 1:** Summary of blood donors.

Donor	Age/Sex	CD14-MLProliferation^a^	HLA–A*02:01/24:02
Healthy donor 1	32/M	++	-/+
Healthy donor 2	36/M	+	+/-
Healthy donor 3	36/M	+	+/+
Healthy donor 4	31/M	+	-/-
Healthy donor 5	32/M	++	-/+
Healthy donor 6	51/M	+	-/+
Healthy donor 7	23/M	++	-/+
Healthy donor 8	50/M	++	NT
Healthy donor 9	36/F	+	NT
Healthy donor 10	35/M	+	NT
Cancer patient 1	60/F	+	-/+
Cancer patient 2	52/M	+	-/+

^*a*^CD14-ML Proliferation: +: more than 100-fold proliferation; ++: more than 1000-fold proliferation.

### Generation of recombinant lentiviruses

A cDNA fragment of human cMYC was obtained by PCR and cloned into the pENTR-TOPO vector (Invitrogen, Carlsbad, CA), and cDNAs for BMI1 and BCL2 were provided by the RIKEN BioResource Center (Tsukuba, Japan). cDNAs for CMVpp65 and MART1/MelanA were generated by gene synthesis (GenScript, Piscataway, NJ). These cDNAs were introduced into a lentivirus vector, pCSII-EF or pCSIIEF-IRES-PuroR, by using the LR Clonase system (Invitrogen). pCSII-EF and the plasmids for lentiviral vector packaging [17], pCMV-VSV-G-RSV-Rev and pCAG-HIVgp, were kindly provided by Dr. H. Miyoshi (Keio University). Plasmid constructs were introduced into 293T cells by lipofection (Lipofectamine 2000, Invitrogen) and 3 days later, the recombinant lentiviruses were recovered from the culture supernatant by centrifugation (50,000 ×*g* for 2 h), as described previously [18].

### Generation of proliferating myeloid lineage cells from monocytes

Monocytes were purified from PBMCs that were isolated from healthy donors using Ficoll-Plaque (GE Healthcare UK, Buckinghamshire, UK) by positive selection with anti-human CD14 MicroBeads (Miltenyi Biotec, Bergisch Gladbach, Germany), and transduced with lentiviral vectors in the presence of polybrene (8 ng/ml; Sigma, St. Louis, MO). The cells were cultured in α-MEM (Wako, Tokyo, Japan) containing 20% FCS (Nichirei Bioscience, Tokyo, Japan), 50 ng/ml of M-CSF (ORF Genetics, Kopavogur, Iceland), and 50 ng/ml of GM-CSF (Prospec, East Brunswick, NJ). After 4–5 weeks, proliferating cells (CD14-ML) appeared [16]. To induce differentiation of CD14-ML into DC, the cells were cultured in the presence of M-CSF (50 ng/ml), GM-CSF (50 ng/ml) and IL-4 (20 ng/ml) for 3 days. To induce the maturation, CD14-ML-DC were stimulated with penicillin-killed *Streptococcus pyogenes* (10 μg/ml, OK432; Chugai Pharmaceutical, Tokyo, Japan) for 2 days.

### Flow cytometric analysis

The following FITC- or PE-conjugated mAbs were purchased from BD Pharmingen (San Diego, CA), eBioscience (San Diego, CA), R&D Systems (Minneapolis, MN), Abcam (Bristol, UK) or Miltenyi Biotec (Bergisch Gladbach, Germany): anti-HLA class II (clone TU39, mouse IgG2a), anti-HLA class I (clone G46-2.6, mouse IgG1), anti-CD80 (clone L307.4, mouse IgG1), anti-CD83 (clone HB15e, mouse IgG1), anti-CD86 (clone FUN-1, mouse IgG1), anti-CD40 (clone 5C3, mouse IgG1), and anti-CD8 (clone T8, mouse IgG1). Mouse IgG2a (clone G155-178), mouse IgG2b (clone 27–35), mouse IgG1 (clone MOPC-21), affinity purified mouse IgM (eBioscience), and rat IgG2a (clone eBR2a) were used as isotype-matched controls. The cell samples were treated with an Fc-receptor-blocking reagent (Miltenyi Biotec) for 10 min, stained with the fluorochrome-conjugated mAb for 30 min, and washed 3 times with PBS containing 2% FCS. The stained cell samples were analyzed on a FACScan (BD Biosciences, Bedford, MA) flow cytometer.

### Quantitation of IL-12p70 production

To analyze the production of IL-12p70 by mo-DC and CD14-ML-DC, the cells were cultured in 96-well flat-bottomed culture plates (1 × 10^5^ cells/200 μl medium/well) in the presence of OK432 (10 μg/ml). After 60 h of culture, the supernatant was collected and the concentration of IL-12p70 was measured using an ELISA kit (eBioscience).

### T cell-proliferation assay

T cells were purified from PBMCs by negative selection with a Pan T-cell isolation kit II (Miltenyi Biotec) and were cultured (3 × 10^4^ cells/well) with graded numbers of X-ray-irradiated (45 Gy) allogeneic stimulator cells (mo-DC, CD14-ML or CD14-ML-DC) in 200 μl AIM-V (Life Technologies, Carlsbad, CA), supplemented with 5% decomplemented human plasma in 96-well round-bottomed culture plates for 5 days. [^3^H]-methylthymidine (247.9 Gbq/mmol) was added to the culture medium (0.037 Mbq/well) in the last 16 h. The uptake of [^3^H]-methylthymidine was measured by scintillation counting. Graded numbers of CD14-ML-DC were pulsed with glutamic acid decarboxylase 65 (GAD65)_111-131_ peptide (LQDVMNILLQYVVKSFDRSTK, 10 μM) for 3 h, X-ray-irradiated (45 Gy), and cultured with GAD65-specific, HLA-DR53-restricted human CD4^+^ T cells (3 × 10^4^ cells/well) [19] in 200 μl AIM-V, supplemented with 5% decomplemented human plasma in 96-well flat-bottomed culture plate for 3 days. The proliferative response of the T cells was measured by incorporation of [^3^H]-methylthymidine. To examine the natural processing of protein antigens, CD14-ML-DC (2 × 10^4^ cells/well) were pulsed with recombinant Glutathion S-transferase (GST)-fused GAD65 protein [20] or GST protein in 96-well flat-bottomed culture plate for 16 h, X-ray-irradiated (45 Gy), and subsequently added to GAD65-specific T cells (3× 10^4^ cells/well) in 200 μl AIM-V, supplemented with 5% human decomplemented plasma. The proliferative response of the T cells was measured by the incorporation of [^3^H]-methylthymidine.

### Generation of cancer antigen-reactive CD8^+^ T cell lines

HLA-A, DRB1, and DPB1 genotyping of blood samples was performed at the HLA Laboratory (Kyoto, Japan) based on a PCR-SSOP (sequence-specific oligonucleotide probe) method. Induction of antigen-specific CD8^+^ T cells was performed as described previously with some modifications [5]. Briefly, CD8^+^ T cells were purified from PBMCs by positive selection with magnetic microbeads (Miltenyi Biotec, Auburn, CA). CD14-ML-DC were used as stimulator cells to induce antigen-specific CD8^+^ T cell lines. CDCA1_351-359_ (KLATAQFKI), KIF20A_809-817_ (CIAEQYHTV), MART1_26-35_ (EAAGIGILTV) and IMP3_515-523_ (NLSSAEVVV) have been identified as HLA-A*02:01-restricted epitopes [5, 6, 21, 22]. CDCA1_56-64_ (VYGIRLEHF), KIF20A_66-75_ (KVYLRVRPLL), LY6K_177-186_ (RYCNLEGPPI) and IMP-3_508–516_ (KTVNELQNL) have been identified as HLA-A*24:02-restricted epitopes [7, 8, 23]. At the beginning of T cell-stimulation culture (day 0) CD14-ML-DC (1 × 10^5^/well) pre-treated with OK432 were pulsed with 10 μg/ml of these 9 or 10-mer peptides for 3 h, X-ray-irradiated (45 Gy), and subsequently cultured with CD8^+^ T cells (2 × 10^6^/well) in 2 ml AIM-V supplemented with 5% decomplemented human plasma and 10 ng/ml recombinant human IL-7 (rhIL-7) in 24-well culture plates. On days 7 and 14, half of the medium was removed from each culture, and fresh medium (1 ml/well) containing irradiated (45 Gy) CD14-ML-DC (not stimulated with OK432; 1 × 10^4^/ well) pre-loaded with the peptides (10 μg/ml) and 10 ng/ml rhIL-7 was added. On day 9, rhIL-2 was added to each well (20 IU/ml). At the end of the culture, the stimulated CD8^+^ T cells were analyzed for specificity by IFN-γ ELISPOT assays and were stained with PE-labeled tetramer of HLA-A*02:01/CDCA1_351-359_, HLA-A*02:01/IMP3_515-523_, HLA-A*24:02/CDCA1_56-64_ or HLA-A*24:02/LY6K_177-186_ peptide complex (MBL, Nagoya, Japan), or with PE-labeled dextramer of the HLA-A*02:01/MART1_26-35_ peptide complex (Immunodex, Copenhagen, Denmark), in combination with FITC-labeled anti-human CD8 mAb (clone T8; Beckman Coulter, Brea, CA), and analyzed by flow cytometry.

### Generation of antigen-reactive CD4^+^ T cell lines

With some modifications, induction of antigen-specific CD4^+^ T cells was performed as described previously [23]. CD4^+^ T cells were purified from PBMCs by positive selection with magnetic microbeads (Miltenyi Biotec, Auburn, CA). CD14-ML-DC were used as APCs to induce antigen-specific CD4^+^ T cells. CDCA1_39-64_ (NPKPEVLHMIYMRALQIVYGIRLEHF), CDCA1_55-78_ (IVYGIRLEHFYMMPVNSEVMYPHL), KIF20A_60-84_ (DSMEKVKVYLRVRPLLPSELERQED), KIF20A_809-833_ (CIAEQYHTVLKLQGQVSAKKRLGTN), LY6K_119-142_ (KWTEPYCVIAAVKIFPRFFMVAKQ) and LY6K_172-191_ (KCCKIRYCNLEGPPINSSVF) have been identified as epitopes for human CD4^+^ T cells in previous studies [23–25]. At day 0, CD14-ML-DC (1 × 10^4^/well) that were pre-treated with OK432 were pulsed with a mixture of the peptides (10 μg/ml each) for 3 h, X-ray-irradiated (45 Gy), and co-cultured with CD4^+^ T cells (3 × 10^4^/ well) in 200 μl AIM-V supplemented with 5% decomplemented human plasma in 96-well flat-bottomed culture plates. At day 7, half of the medium was removed and replaced with fresh medium (100 μl/well) containing irradiated (45 Gy) CD14-ML-DC (not stimulated with OK432; 1 × 10^4^/well) that were pre-pulsed with peptides (10 μg/ml) and rhIL-7 was added (5 ng/ml). At day 9, rhIL-2 was added to each well (10 IU/ml). At day 14, CD4^+^ T cells in each well were analyzed for peptide-specific reactivity based on IFN-γ ELISPOT assays. The T cells showing a specific response to the cognate peptide were transferred to 24-well plates and restimulated at weekly intervals with irradiated CD14-ML-DC (not stimulated with OK432; 1 × 10^5^/ well) loaded with the peptide, and subsequently added with medium that was supplemented with rhIL-2 (20 IU/ml) and rhIL-7 (5 ng/ml). At day 28, the stimulated CD4^+^ T cells were harvested, washed, and analyzed for peptide-specific production of IFN-γ by ELISPOT assay. To decide the restriction HLA molecules, anti-HLA-DP mAb (B7/21; Abcam), anti-HLA-DQ mAb (SPV-L3; Abcam) or anti-HLA-DR mAb (L243; Bio Legend) was added (all mAbs 5 μg/ml) to the stimulator cells (autologous PBMC) in ELISPOT plates. After incubation for 1 h, peptides and T cells were added. Spot numbers were counted after 16 h of co-culture.

### Generation of CD14-ML-DC expressing antigenic proteins

CD14-ML were transduced with lentiviral vectors encoding for CMVpp65 or MART1/MelanA in the presence of polybrene (8 ng/ml). The cells were cultured in α-MEM containing 20% FCS, M-CSF (50 ng/ml), and GM-CSF (50 ng/ml). Two days after the transduction with the lentiviral vectors, cells were cultured in the presence of puromycin (10 μg/ml) for 2 weeks. Expression of the antigenic proteins in the modified CD14-ML was examined by flow cytometric analysis. Subsequently, antigen-expressing CD14-ML (CD14-ML/CMV or CD14-ML/MART1) were added with IL-4 (20 ng/ml) and allowed to differentiate into DC (CD14-ML-DC/CMV or CD14-ML-DC/MART1).

### Stimulation of CD8^+^ T cells with CD14-ML-DC/CMV

CD14-ML-DC/CMV were stimulated with TNF-α (5 ng/ml) for 2 days, irradiated, and plated in 24-well culture plates (1 × 10^5^ cells/well). Autologous peripheral blood CD8^+^ T cells were added to the wells (2 × 10^6^ cells/well). The cells were cultured in AIM-V medium, containing 5% human decomplemented plasma and rhIL-7 (10 ng/ml). rhIL-2 (20 IU/ml) was added on day 2. On day 9, the cells were harvested, stained with a PE-labeled tetramer of the HLA-A*24:02/CMVpp65_341-349_ (QYDPVAALF) complex (MBL, Nagoya, Japan) in combination with a FITC-labeled anti-human CD8 mAb, and analyzed by flow cytometry. To analyze IFN-γ production by CMV-specific T cells, CD8^+^ T cells (1 × 10^4^ cells/well) were co-cultured with CD14-ML/CMV or C1R-A24 (HLA-A*24:02-expressing C1R cells) (1 × 10^4^ cells/well) that were pre-pulsed with the HLA-A*24:02-binding CMV pp65_341-349_ peptide [26]. After 16 h, the culture was terminated and IFN-γ producing cells were counted using an ELISPOT assay.

### Stimulation of CD8^+^ T cells with CD14-ML-DC/MART1

CD14-ML-DC/MART1 were stimulated with OK432, irradiated, and co-cultured with autologous CD8^+^ T cells (2 × 10^6^ cells/well) in 2 mL AIM-V, supplemented with 5% human decomplemented plasma and rhIL-7 (10 ng/ml) in a 24-well culture plate. CD14-ML-DC/MART1 and rhIL-7 were added on days 7 and 14, and rhIL-2 (20 IU/ml) was added on days 9 and 16. On day 21, the CD8^+^ T cells were harvested, stained with a PE-labeled dextramer of the HLA-A*02:01/MART1 peptide complex in combination with a FITC-labeled anti-human CD8 mAb, and analyzed by flow cytometry. To analyze IFN-γ production by ELISPOT assay, CD8^+^ T cells (1 × 10^4^ cells/well) were co-cultured with autologous CD14-ML-DC/MART1 or HLA-A*02:01-positive T2 cells (1 × 10^4^ cells/well) that were pre-pulsed with MART1_26-35_ peptide for 16 h.

## Results

### Improved method for generation of CD14-ML

We previously reported induction of proliferating CD14^+^ monocytes by lentivirus-mediated introduction of cMYC and BMI1, resulting in the generation of CD14-ML [16]. Nonetheless, there was variation in proliferation efficiency among blood donors, and we could not induce the proliferation of monocytes derived from 3 out of the 12 blood donors in the previous study [16]. In the current study, we tried to improve the efficiency in generating CD14-ML. For this purpose, we searched for another factor to add to the 2 genetic factors already in place. We tested several factors that might help or enhance cell proliferation, including BCL2, LYL1, BCL-XL, MLLT1, MEIS1, HOXA9, E2F2, E4F1, PHC1, PBX1, CDT1, MLF1, Cyclin D2, MYB, SOX2, CBX5, AURKB, and BUB1 (Figure A in S1 Fig). As a result, we found that the introduction of BCL2 or LYL1 with the previously used factors, cMYC and BMI1, markedly improved the efficiency of proliferation induction (Figure B in S2 Fig, S1 Table).

**Fig 1:**
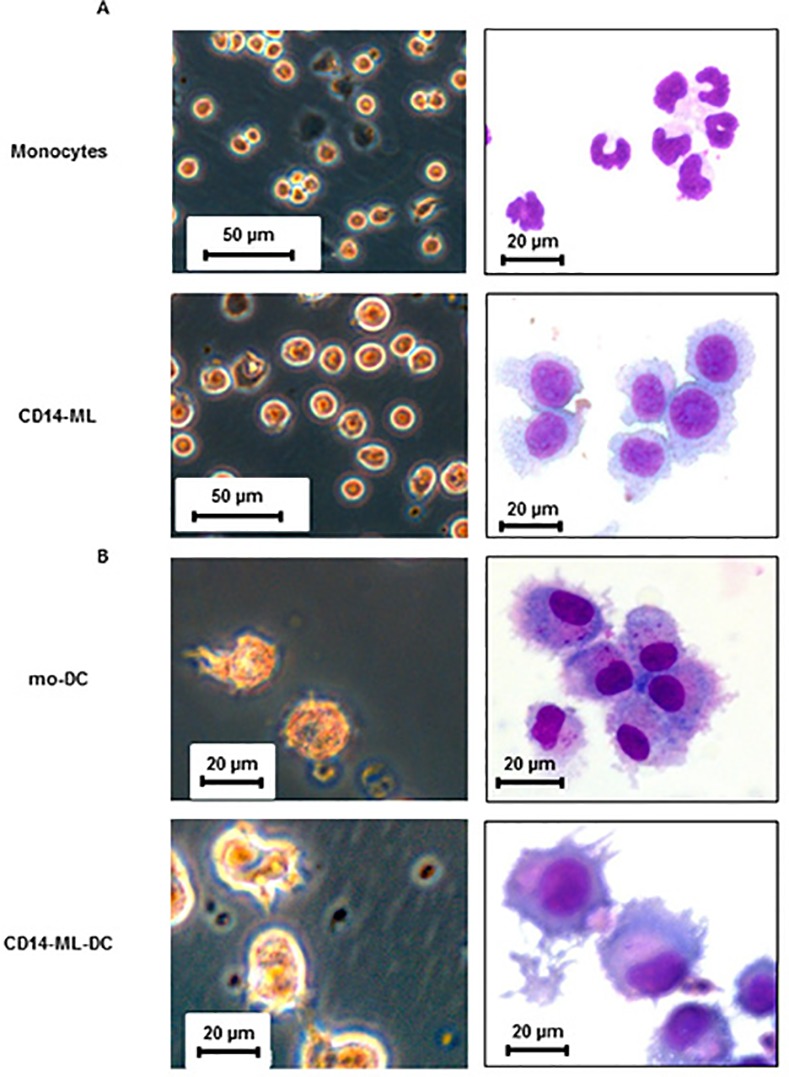
Morphology of CD14-ML and CD14-ML-DC generated by the current procedure. (A) Phase-contrast images of live cells (left) and cytospin samples stained with May–Grünwald Giemsa (right) of the human monocytes and monocyte-derived myeloid cell lines (CD14-ML) are shown. (B) Morphology of OK432-stimulated mo-DC and CD14-ML-DC are shown. mo-DC and CD14-ML-DC were stimulated with OK432 for 2 days and subjected to microscopic analysis. The data are representative of 2 experiments.

Four to five weeks after introducing cMYC, BMI1, plus BCL2, we observed proliferation of cells. Monocyte samples from all the donors exhibited significant proliferation, provided that they were freshly prepared but not freeze-thawed. The samples used in the current study included samples from that we could not establish CD14-ML with the introduction of cMYC and BMI1 only. Although the duration, rate, and the magnitude of proliferation still varied among the donors, there was at least a 100-fold increase in cell number attained with the current method (Table 1). Similar to the CD14-ML generated with the previous method, the proliferation of CD14-ML using the current method was dependent on the presence of both M-CSF and GM-CSF [16]. As shown in Fig 1A, CD14-ML were larger than monocytes and possessed round nuclei instead of monocyte-like, irregularly shaped nuclei.

### DC differentiation of CD14-ML that were generated using the improved procedure

**Fig 2:**
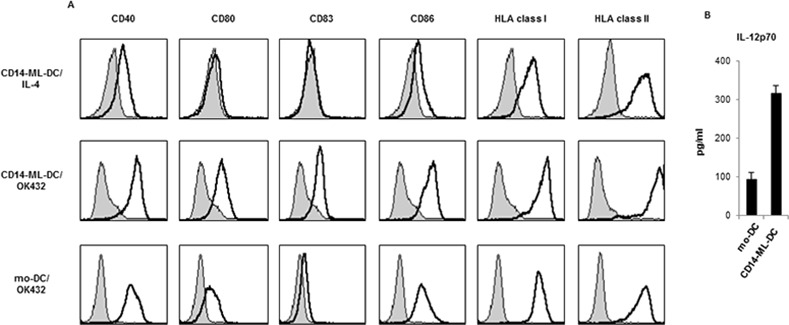
Cell surface molecules of CD14-ML-DC and the production of IL-12p70 by CD14-ML-DC. (A) CD14-ML-DC, before and after treatment with OK432, were analyzed for the expression of CD40, CD80, CD83, CD86, HLA class I and HLA class II. As a control, results of the analysis of OK432-stimulated mo-DC (monocyte-derived DC) are also shown. The staining profiles of the specific mAbs (black lines) and isotype-matched control mAbs (gray area) are shown. (B) CD14-ML-DC and mo-DC were cultured in 96-well flat-bottomed culture plates (1×10^5^ cells/200 μl medium/well) in the presence OK432 (10 μg/ml). After 60 h, the concentration of IL-12p70 in the culture supernatant was measured by ELISA. The data are representative of 2 experiments.

In order to let CD14-ML differentiate into DC, we added IL-4. After 3 days, we added OK432 (penicillin-killed *Streptococcus pyogenes*), a potent maturation inducer for human monocyte-derived DC (mo-DC) [27–29]. Similar to the CD14-ML generated by the previous method, CD14-ML generated by the current procedure also differentiated into CD14-ML-DC with typical DC-like morphology and protrusions (Fig 1B). Expression of CD80, CD86, CD83, CD40, HLA class I, and HLA class II was detected in CD14-ML-DC and their expression was enhanced by stimulation with OK432 (Fig 2A). The levels of expression for these molecules were almost equal to that of mo-DC (Fig 2A). Under the stimulation with OK432, CD14-ML-DC produced higher amounts of IL-12p70 than mo-DC (Fig 2B). Collectively, CD14-ML that was generated by the current method differentiated into CD14-ML-DC and possessed the phenotype of fully mature DC.

### T cell stimulation and antigen presentation by CD14-ML-DC

**Fig 3:**
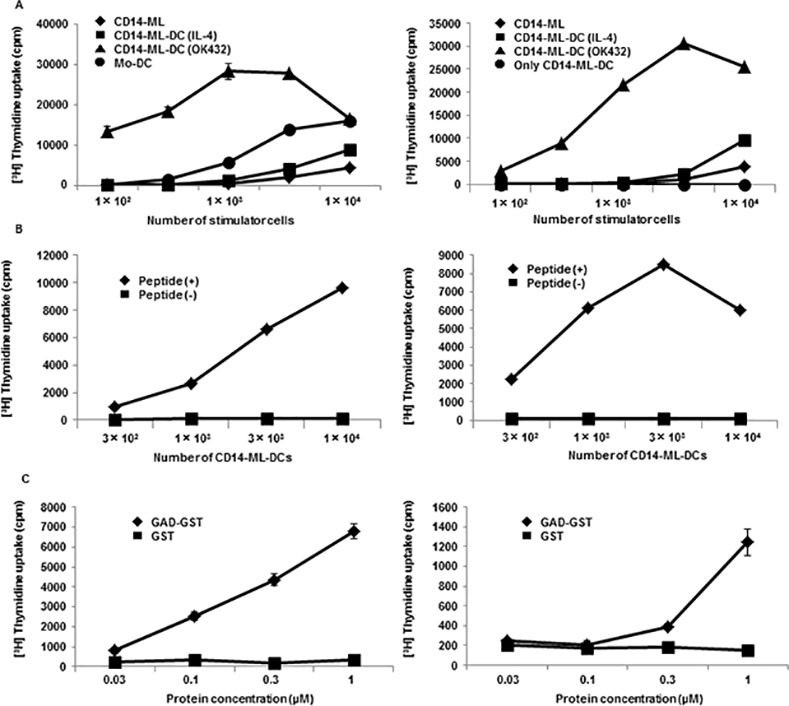
T-cell stimulation and antigen presentation by CD14-ML-DC. (A) CD14-ML (rhombuses), CD14-ML-DC stimulated with IL-4 (squares), OK432 (triangles), or mo-DC (circles) were irradiated and co-cultured with allogeneic peripheral blood T cells (4×10^4^ cells/well) or alternatively, only CD14-ML-DC (circles) were irradiated and cultured in a 96-well round-bottomed culture plates for 5 days. T cell proliferation was measured according to [^3^H]-methylthymidine-uptake in the last 16 h. The experiments were conducted on CD14-ML derived from 2 different donors. (B) The indicated numbers of CD-14-ML-DC were loaded with GAD65_111-131_ peptide (rhombuses) or those left unloaded (squares), X-ray-irradiated, and co-cultured with GAD65-specific HLA-DR53-restricted clonal human CD4^+^ T cells (3×10^4^ cells/well) for 3 days. The proliferative response of the T cells was measured by incorporation of [^3^H]-methylthymidine in the last 16 h of culture. The experiments were conducted on CD14-ML derived from 2 different donors. (C) CD14-ML-DC were pulsed with recombinant GST-fused GAD65 protein or GST protein for 16 h, X-ray-irradiated, and subsequently added to GAD65-specific T cells. The proliferative response of the T cells was analyzed. The experiments were conducted on CD14-ML derived from 2 different donors.

We examined the ability of CD14-ML-DC to stimulate naive T cells with a primary allogeneic T cell stimulation assay. Compared to CD14-ML and mo-DC, CD14-ML-DC induced a higher magnitude of proliferation in the allogeneic T cells (Fig 3A). Treatment with OK432 further enhanced the T cell stimulation capacity of CD14-ML-DC.

Next, we examined whether CD14-ML-DC were able to present an antigenic peptide in the context of HLA class II. We used a previously established clone of GAD65_111-131_-specific DR53 (DRB4*01:03)-restricted human CD4^+^ T cells as the responder. CD14-ML-DC derived from a DR53-positive donor were pulsed with a GAD65 peptide and cultured with the T cell clone (Fig 3B). The T cells showed specific proliferative responses, indicating that CD14-ML-DC were able to present an antigenic peptide in the context of HLA class II to stimulate the T cells.

We examined the processing of protein antigens. CD14-ML-DC were cultured in the presence of recombinant GST-fused GAD65 protein or GST protein for 16 h before the addition of GAD65-specific T cells. The GAD65-specific T cells proliferated in a dose-dependent manner, indicating that CD14-ML-DC were able to process a protein antigen and present an antigenic peptide (Fig 3C).

### Induction of antigen-specific CD8^+^ T cells from healthy donors by CD14-ML-DC

**Fig 4:**
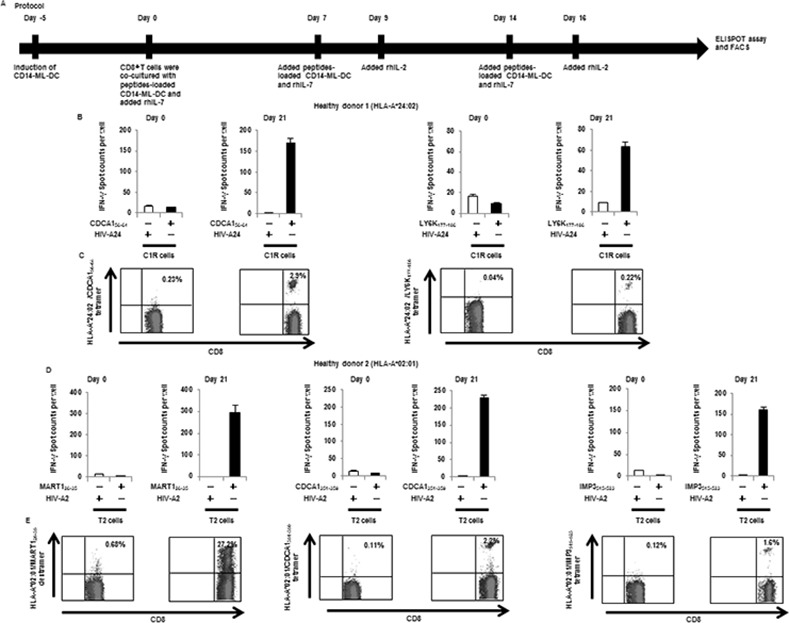
Induction of CD8^+^ T cell lines that are reactive to cancer antigens by CD14-ML-DC. (A) Protocol for the induction of cancer antigen-specific CD8^+^ T cells by CD14-ML-DC. In order to generate CD14-ML-DC, we added IL-4 to CD14-ML. After 3 days, we added OK432. CD14-ML-DC were pulsed with peptides for 3 h, X-ray-irradiated (45 Gy), and subsequently mixed with autologous CD8^+^ T cells. Cells were cultured with rIL-7 (10 ng/ml) in AIM-V with 5% human decomplemented plasma. On days 7 and 14, the T cells were restimulated with the autologous peptide-pulsed CD14-ML-DC and on days 9 and 16, and were supplemented with rIL-2 (20 IU/ml). CD14-ML-DC were prepared each time, and we only added IL-4 (did not add OK432). IFN-γ ELISPOT assay and flow cytometry were performed after 6 or 7 days from the third round of peptide stimulation. (B, C) Peripheral blood CD8^+^ T cells were obtained from a HLA-A*24:02-positive healthy donor (healthy donor 1) and were co-cultured with 4 peptides (CDCA1_56-64_, KIF20A_66-75_, LY6K_177-186_ and IMP-3_508–516_)-loaded autologous CD14-ML-DC. (B) On day 21, the number of IFN-γ producing CD8^+^ T cells were analyzed by ELISPOT assay (Day 21). The results of the T cells before stimulation culture are also shown (Day 0). The HIV-peptide was used as a control peptide. (C) On day 21, the T cells were recovered and stained with anti-CD8 mAb and the HLA-A*24:02/CDCA1_56-64_ or HLA-A*24:02/LY6K_177-186_ tetramer. The numbers in the figure indicate the percentage of the CD8^+^ T cells that were positively stained with the tetramer of the HLA-peptide complex (Day 21). The results of the T cells before stimulation culture are also shown (day 0). (D, E) A similar experiment as in (B, C) was done with the cells obtained from a HLA-A*02:01-positive donor (healthy donor 2). We used 4 peptides (CDCA1_351-359_, KIF20A_809-817_, MART1_26-35_ and IMP3_515-523_) for the stimulation of the T cells. (D) The number of IFN-γ producing CD8^+^ T cells was analyzed by ELISPOT assay. (E) The T cells were recovered and stained with an anti-CD8 mAb and a HLA-A*02:01/MART1_26-35_ dextramer, HLA-A*02:01/CDCA1_351-359_ tetramer or HLA-A*02:01/IMP3_515-523_ tetramer. The numbers in the figure indicate the percentage of the CD8^+^ T cells that were positively stained with the dextramer or tetramer of HLA-peptide complex.

We tried to prime cancer antigen-specific CD8^+^ T cells by *in vitro* stimulation with antigenic peptide-loaded CD14-ML-DC, according to the culture protocol shown in Fig 4A. Peripheral blood CD8^+^ T cells derived from HLA-A*02:01 or HLA-A*24:02-positive healthy donors were co-cultured with autologous CD14-ML-DCs pre-loaded with a mixture of 4 kinds of previously identified cancer antigen-derived peptides (CDCA1_56-64_, KIF20A_66-75_, LY6K_177-186_ and IMP-3_508–516_ for HLA-A*24:02; CDCA1_351-359_, KIF20A_809-817_, MART1_26-35_ and IMP3_515-523_ for HLA-A*02:01). Stimulation of T cells with peptide-loaded DC was repeated once a week for 3 weeks. On day 21, the T cells were harvested and analyzed for their reactivity to the individual peptides using ELISPOT analysis, with HLA-A*24:02-positive C1R cells or HLA-A*02:01-positive T2 cells as stimulators.

Of the T cells from healthy donors (1 positive for HLA-A*24:02), T cells reactive to CDCA1_56-64_ and LY6K_177-186_ were induced at the end of 3 weeks of induction culture (Fig 4B and 4C). Staining with a tetramer of HLA-A*24:02/peptide complex verified the expansion of specific T cells during the 3 weeks of stimulation culture. Similarly, T cells reactive to CDCA1_351-359_, MART1_26-35_, and IMP3_515-523_ were induced from peripheral blood CD8^+^ T cells from the healthy donor 2 that was positive for HLA-A*02:01 (Fig 4D and 4E). These results suggest that HLA class I-restricted peptide-loaded CD14-ML-DC were able to stimulate specific CD8^+^ T cells in autologous CD8^+^ T cell populations and induce their expansion in the same way as mo-DC.

### Stimulation of cancer antigen-specific CD4^+^ T cells by CD14-ML-DC

**Fig 5:**
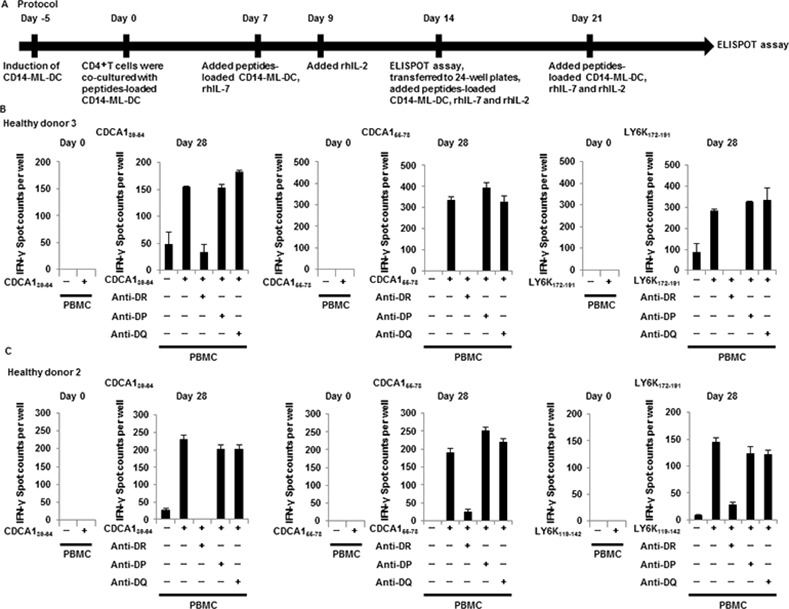
Induction of CD4^+^ T cell lines that are reactive to cancer antigens by CD14-ML-DC. (A) Protocol for the induction of cancer antigen-specific CD4^+^ T cells by CD14-ML-DC. In order to generate CD14-ML-DC, we added IL-4 to CD14-ML. After 3 days, we added OK432. CD14-ML-DC were pulsed with a mixture of 6 peptides (CDCA1_39-64_, CDCA1_55-78_, KIF20A_60-84_, KIF20A_809-833_, LY6K_119-142_ and LY6K_172-191_) for 3 h, X-ray-irradiated (45 Gy), and subsequently mixed with autologous CD4^+^ T cells in AIM-V with 5% human decomplemented plasma. On day 7, the T cells were restimulated with the autologous peptide-pulsed CD14-ML-DC and supplemented with rIL-7 (5 ng/ml). After two days, these cultures were supplemented with rIL-2 (10 IU/ml). CD14-ML-DC were added with only IL-4 (did not add OK432). On day 14, the stimulated CD4^+^ T cells in each well were analyzed for specificity in IFN-γ ELISPOT assays. The T cells showing a specific response to the cognate peptide were transferred to 24-well plates and restimulated with the autologous peptide-pulsed CD14-ML-DC, and subsequently supplemented with rhIL-7 (5 ng/ml) and rhIL-2 (20 IU/ml). On day 21, the T cells were restimulated with the autologous peptide-pulsed CD14-ML-DC and supplemented with rhIL-7 and rhIL-2. IFN-γ ELISPOT assays were performed after 6 or 7 days from the fourth round of peptide stimulation. (B, C) After the stimulation (more than three times), the number of CD4^+^ T cells reacting to each peptide was analyzed with an IFN-γ ELISPOT assay (Day 28). The results of the T cells before stimulation culture are shown (Day 0). Dimethyl sulfoxide was used as a control. The results for the healthy donor 3 (B) and donor 2 (C) are shown.

We next examined CD14-ML-DC on their capacity to induce antigen-specific CD4^+^ T cells. We used six kinds of cancer antigen-derived peptides (CDCA1_39-64_, CDCA1_55-78_, KIF20A_60-84_, KIF20A_809-833_, LY6K_119-142_ and LY6K_172-191_) that were previously identified as HLA-DR-restricted epitopes [23–25]. CD4^+^ T cells isolated from the PBMCs of healthy donors were stimulated with autologous CD14-ML-DC that were pulsed with the peptide mixture. After at least three rounds of stimulation, peptide-specific responses of the resultant CD4^+^ T cells were examined by an IFN-γ ELISPOT assay with autologous PBMCs as stimulator cells (culture schedule shown in Fig 5A).

Resultant CD4^+^ T cell lines from healthy donor 3 reacted to CDCA1_39-64_, CDCA1_55-78_ and LY6K_172-191_ in an HLA-DR-dependent manner (Fig 5B). Similar results were obtained for healthy donor 2 (Fig 5C). These results indicate that peptide-loaded CD14-ML-DC were able to induce antigen-specific CD4^+^ T cells in an *in vitro* stimulation culture.

### T cell stimulation by CD14-ML-DC established from cancer patients

Monocytes obtained from cancer patients are often less viable than those from healthy donors. In such cases, the generation of DC from monocytes has been reported to be very difficult [30]. Thus, it would be very useful if CD14-ML could be established not only from healthy donors but also from cancer patients. We tried to establish CD14-ML from monocytes obtained from HNC patients. As a result, we successfully established CD14-ML from monocytes obtained from the 2 cancer patients (cancer patient 1 and 2), and the CD14-ML were able to differentiate into CD14-ML-DC after stimulation with IL-4.

**Fig 6:**
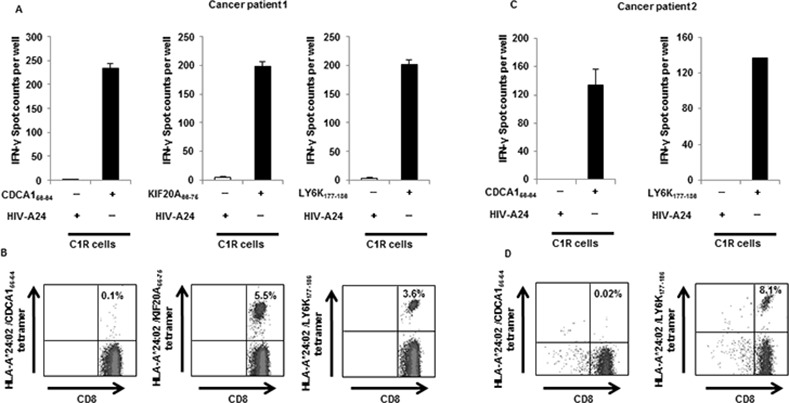
Induction of CD8^+^ T cell lines that are reactive to cancer antigens by CD14-ML-DC obtained from HNC patients. Peripheral blood CD8^+^ T cells obtained from HLA-A*24:02-positive HNC patients were co-cultured with autologous CD14-ML-DC pre-loaded with a peptide mixture (CDCA1_56-64_, KIF20A_66-75_, LY6K_177-186_ and IMP-3_508–516_) to induce T cell lines that were reactive to the peptides under a schedule similar to that shown in Fig 4A. On days 7 and 14, the CD8^+^ T cells were re-stimulated with peptide-loaded CD14-ML-DC. (A, C) On day 21, the number of CD8^+^ T cells responding to the peptides were analyzed by an IFN-γ ELISPOT assay. An HIV-peptide was used as a control peptide. (B, D) On day 21, the T cells were recovered and stained with an anti-CD8 mAb and a HLA-A*24:02/CDCA1_56-64_, HLA-A*24:02/KIF20A_66-75_ or HLA-A*24:02/LY6K_177-186_ tetramer. The numbers in the figure indicate the percentage of the CD8^+^ T cells that were positively stained with the tetramer from the HLA-peptide complex. The results for the cancer patient 1 (A, B) and the cancer patient 2 (C, D) are shown.

We examined the capacity of CD14-ML-DC to induce antigen-specific CD8^+^ T cell lines. Stimulation of CD8^+^ T cells by peptide antigen-loaded autologous CD14-ML-DC in induction cultures was carried out using the same protocols as those that were used for cell samples from healthy donors. To analyze the induction of antigen-specific T cells, resulting CD8^+^ T lines were subjected to IFN-γ ELISPOT analysis and stained with an HLA/peptide tetramer. The response of CD8^+^ T cells to CDCA1_56-64_, LY6K_177-186_, and KIF20A_66-75_ was observed for cancer patients (Fig 6A–6D).

**Fig 7:**
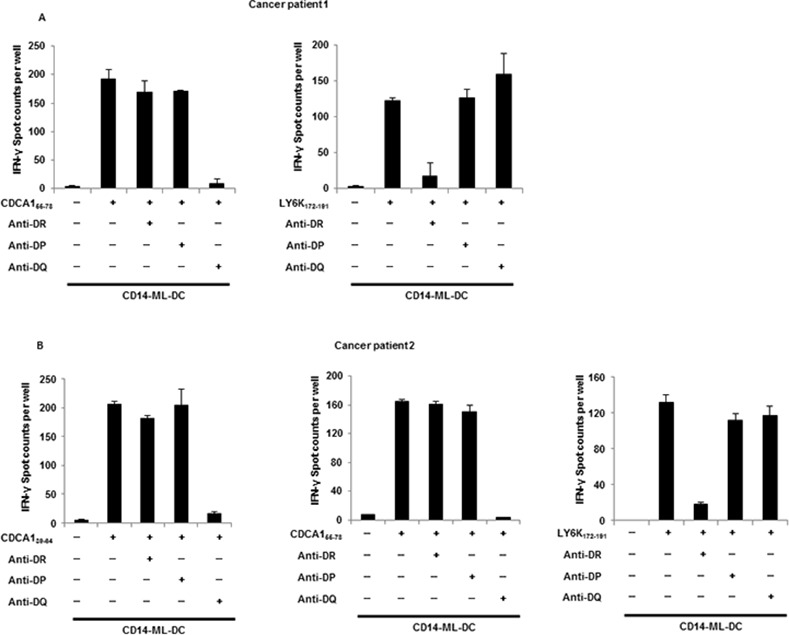
Induction of CD4^+^ T cell lines that are reactive to cancer antigens by CD14-ML-DC obtained from HNC patients. CD4^+^ T cells isolated from PBMCs of HNC patients were stimulated with CD14-ML-DC that were pulsed with a mixture of 6 kinds of peptides (CDCA1_39-64_, CDCA1_55-78_, KIF20A_60-84_, KIF20A_809-833_, LY6K_119-142_ and LY6K_172-191_). After more than three rounds of stimulation, the number of CD4^+^ T cells that reacted to each peptide was analyzed by ELISPOT assay. CD14-ML-DC were used as stimulators in the assay, because only a few amount of blood samples could be obtained from cancer patients. The results for cancer patient 1 (A) and cancer patient 2 (B) are shown.

Similarly, we tried to induce of cancer antigen-specific CD4^+^ T cell lines by using CD14-ML-DC derived from cancer patients. The cancer patient 1-derived CD4^+^ T cell lines that were harvested from the induction culture responded to the CDCA1_55-78_ and LY6K_172-191_ peptides in an HLA-DQ or HLA-DR-restricted manner (Fig 7A). The cancer patient 2-derived CD4^+^ T cell lines that were harvested from the induction culture responded to the CDCA1_39-764_, CDCA1_55-78_, and LY6K_172-191_ peptides in an HLA-DQ or HLA-DR-restricted manner (Fig 7B).

Collectively, the generation of CD14-ML-DC derived from monocytes of cancer patients was feasible, and the cancer patient-derived CD14-ML-DC were sufficiently potent to induce antigen-specific T cell responses.

### Genetic modification of CD14-ML to generate CD14-ML-DC expressing CMVpp65

We demonstrated the establishment of antigen-specific CD4^+^ or CD8^+^ T cell lines by *in vitro* stimulation with CD14-ML-DC that were pre-loaded with synthetic peptides (Figs 4 and 5). We next generated genetically modified CD14-ML-DC expressing antigenic proteins by taking advantage of the proliferative capabilities of CD14-ML.

**Fig 8:**
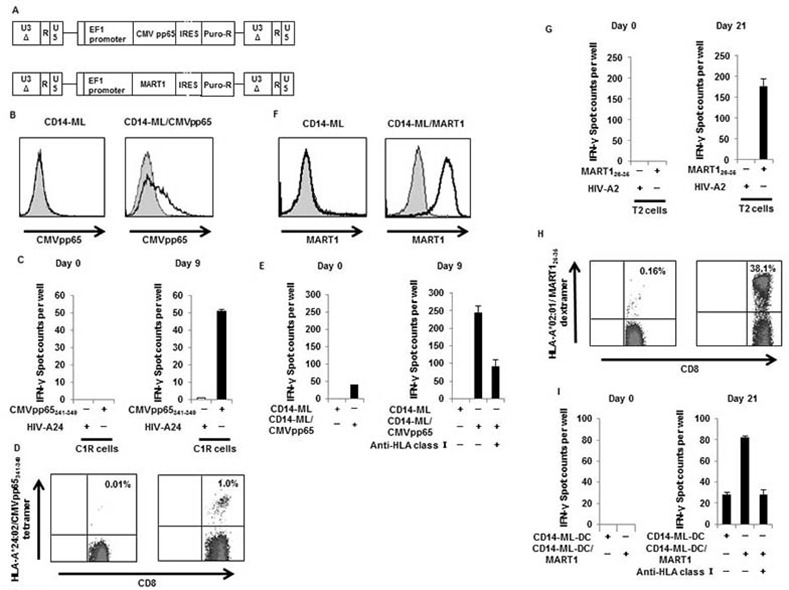
Induction of CD8^+^ T cell lines that are reactive to antigens by CD14-ML-DC that express antigenic proteins. (A) The lentivirus constructs for CMVpp65 (EF-CMV-IP) and MART1 (EF-MART1-IP) are shown. CD14-ML were transduced with the lentivirus vector and cultured in the presence of puromycin (5 μg/ml) to select and expand the cell population carrying the transgene, resulting in the generation of CD14-ML/CMV and CD14-ML/MART1. (B) Expression of CMVpp65 by CD14-ML/CMV was analyzed by flow cytometric analysis. The staining profiles of the specific mAb (black lines) and isotype-matched control mAb (gray area) are shown. (C) CD14-ML-DC/CMV derived from a HLA-A*24:02-positive healthy donor were cultured with autologous CD8^+^ T cells. On day 9, the number of T cells reactive to the CMVpp65_341-349_ peptide was analyzed by ELISPOT assay. The HIV-peptide was used as a control peptide. The results of the T cells before stimulation culture are shown (Day 0). (D) On day 9, the T cells were recovered and stained with an anti-CD8 mAb and a tetramer of HLA-A*24:02/CMVpp65_341-349_ complex. The numbers in the figure indicate the percentage of the CD8^+^ T cells positively stained with the tetramer of the HLA-peptide complex. The results of the T cells before stimulation culture are also shown (Day 0). (E) CD8^+^ T cells obtained from an HLA-A*24:02-negative healthy donor were co-cultured with autologous CD14-ML-DC/CMV. On day 9, the number of IFN-γ producing CD8^+^ T cells was analyzed by ELISPOT assay, using CD14-ML and CD14-ML/CMV as stimulators. The results of the T cells before stimulation culture are also shown (Day 0). (F) Expression of MART1 by CD14-ML/MART1 was analyzed by flow cytometric analysis. The staining profiles of the specific mAbs (black lines) and isotype-matched control mAbs (gray area) are shown. (G) CD8^+^ T cells obtained from an HLA-A*02:01-positive healthy donor were co-cultured with autologous CD14-ML-DC/MART1 cells. On day 21, the frequency of CD8^+^ T cells reactive to MART1_26-35_ was analyzed by ELISPOT assay. The HIV-peptide was used as a control peptide. The results of the T cells before stimulation culture are also shown (Day 0). (H) On day 21, the T cells were recovered and stained with an anti-CD8 mAb and HLA-A*02:01/MART1_26-35_ dextramer. The numbers in the figure indicate the percentage of the CD8^+^ T cells that were positively stained with the dextramer of the HLA-peptide complex. The results of the T cells before stimulation culture are also shown (Day 0). (I) CD8^+^ T cells obtained from an HLA-A*02:01-negative healthy donor were co-cultured with autologous CD14-ML-DC/MART1. On day 21, the frequency of CD8^+^ T cells reactive to MART1 was analyzed by ELISPOT assay, using CD14-ML-DC and CD14-ML-DC/MART1 as stimulators. The results of the T cells before stimulation culture are also shown (Day 0).

We transduced CD14-ML derived from an HLA-A*24:02-positive donor with a lentivirus vector for CMVpp65, including an IRES (internal ribosomal entry site)-puromycinacetyltransferase cassette (Fig 8A). The cells were cultured for 2 weeks in the presence of puromycin to select and expand cells expressing the protein. After that, the expression of CMVpp65 in the resultant CD14-ML (CD14-ML/CMV) was confirmed by flow cytometric analysis (Fig 8B). We added IL-4 to CD14-ML/CMV-derived from an HLA-A*24:02-positive donor to generate CD14-ML-DC/CMV, and used them as a stimulator for the induction of CMV-specific CD8^+^ T cell lines from the autologous peripheral blood CD8^+^ T cells. To examine CMV-specific responses, we examined the IFN-γ-producing cells after overnight co-culture with C1R cells that were pulsed with HLA-A*24:02-restricted a CMVpp65-derived epitope (CMVpp65_341-349_). The results shown in Fig 8C indicate the successful induction of CD8^+^ T cell lines reactive to CMVpp65_341-349_. CMVpp65_341-349_-reactive CD8^+^ T cells were also detected by staining with the tetramer (Fig 8D).

Next, we tried to generate a CMV-pp65-reactive CD8^+^ T cell line from another blood donor who is homozygous for HLA-A*26:01 (healthy donor 4). No information on CMV-derived epitopes is available for this HLA-A allele. Generation of CMVpp65-expressing CD14-ML-DC and co-culture with autologous CD8^+^ T cells were done by the same procedure described above. To detect reactivity to CMVpp65, the resulting T cells were co-cultured with autologous CD14-ML with or without the transgene for CMVpp65. As shown in Fig 8E, the response to pp65 protein was observed in this analysis. The T cells stimulated with CD14-ML-DC/CMVpp65 were further activated, and compared to T cells before stimulation culture. The response was blocked by the addition of an anti-HLA class I blocking antibody, indicating that the response was restricted by HLA class I. Although we have not identified the epitope for this donor, it was experimentally demonstrated that stimulation of antigen-specific CD8^+^ T cells is feasible regardless of whether information on the epitope is available or not.

### CD14-ML-DC expressing MART1/Melan-A

We next generated CD14-ML-DC expressing MART1/Melan-A by a similar method as above. Expression of the MART1 protein in the resulting CD14-ML-DC (CD14-ML-DC/MART1) was confirmed by staining with a specific antibody and subsequent flow cytometric analysis (Fig 8F).

CD14-ML-DC/MART1 were generated from 2 blood donors, one (healthy donor 2) who was positive for HLA-A*02:01 and another (healthy donor 4) who was negative for HLA-A*02:01. Both CD14-ML-DC/MART1 were co-cultured with autologous peripheral blood CD8^+^ T cells. The CD8^+^ T cells were stimulated with the CD14-ML-DC/MART1 once a week, and the culture was continued for 3 weeks. On day 21, the cultured T cells were analyzed for their reactivity to MART1.

The cultured T cells from the HLA-A*02:01-positive donor were co-cultured with HLA-A*02:01 expressing T2 cells loaded with known HLA-A*02:01-restricted epitope (MART1_26-35_). The results shown in Fig 8G indicate the induction of the T cells reactive to the HLA-A*02:01-restricted epitope. As shown in Fig 8H, 38.1% of the T cells were positively stained with the dextramer of HLA-A*02:01/MART1_26-35_ complex. MART-1 specific T cells also reacted to an HLA-A*02:01^+^ MART-1-expressing melanoma cell line SK-MEL-5 [31] to produce IFN-γ, indicating that the T cells recognized not only epitope-pulsed Target cells but also cancer cells naturally expressing the antigen (S3 Fig).

Recovered CD8^+^ T cells that were derived from an HLA-A*02:01-negative donor were co-cultured with autologous CD14ML-DC/MART1 or autologous CD14-ML-DC. The results shown in Fig 8I indicate that the T cells responded to MART1, and that the response was HLA-class I-restricted.

Collectively, we can readily generate functional DC expressing antigenic proteins based on the CD14-ML technology. More importantly, by using the antigen-expressing DC, we can induce antigen-specific T cells even if information on the HLA-restricted epitopes is not available.

## Discussion

The present study is based on our previous observation that lentiviral vector-mediated introduction of cMYC and BMI1 induced proliferation of human peripheral blood monocytes [16]. In that study, we did not succeed in generating CD14-ML from monocytes derived from some of the donors. One of the purposes of the current study was to find a way to improve the efficiency of the CD14-ML induction. We assumed that differences in the expression of some factor related to cell proliferation or anti-senescence would explain the variation among the monocytes from different donors, and that we could improve the efficiency of the method by adding additional factors.

In order to find factors to add, we tested several genes related to cell-proliferation, anti-apoptosis, or anti-senescence, including BCL2, LYL1, MEIS1 and other various genes. As a result, we found that the introduction of BCL2 or LYL1 along with cMYC and BMI1 significantly improve efficiency. By the currently improved procedure, we could induce the proliferation of monocytes derived from all healthy individuals and cancer patients, provided that the monocytes were freshly prepared.

We comparatively analyzed the expansion of monocytes subject to old and new methods for CD14-ML generation. In the experiments, we used monocyte samples obtained from two donors (Figure B in S2 Fig). For one sample, vigorous proliferation was induced by the both method and the magnitude of amplification induced by the new method was somehow greater than that by the old one. For another cell sample, significant cell amplification was achieved only by the new method. Features of CD14-ML generated by the current method is indistinguishable from those generated by the original method in terms of morphology, cell surface molecules, dependency on GM-CSF and M-CSF, and capacity to differentiate into DC (Figs 1 and 2).

Critical issue may be the risk of malignancy accompanying clinical use of cells transduced with cMYC, BMI1 plus BCL2 by lentivirus. Although CD14-ML cease to proliferate in several days after addition of IL-4, we cannot totally rule out the risk of development of malignancy. As shown in Fig 3A, proliferative capacity of CD14-ML-DC was completely inactivated by irradiation at 45 Gy. The data suggest that we can avoid the risk of malignancy development by irradiation of CD14-ML-DC before the administration to patients.

Another purpose of the current study is to evaluate the possibility of generating genetically modified CD14-ML-DC expressing antigenic proteins and to examine their ability to induce antigen-specific T cells in culture. In order to generate DC expressing whole antigenic proteins, we introduced lentiviral vectors for the antigenic proteins, CMVpp65 or MART1/MelanA, into CD14-ML. The vectors were designed to drive the expression of the antigenic proteins by the EF1 promoter and contained an IRES-puromycinacetyltransferase in the downstream region of the cDNA for the antigenic protein. We can efficiently select CD14-ML expressing the antigen by culture in the presence of puromycin. Indeed, the selected cells were mostly positive for the expression of the transgene-derived proteins (Fig 8B and 8F). Subsequently, the CD14-ML were subjected to differentiation culture in order to generate CD14-ML-DC. As shown in Fig 8, *in vitro* stimulation of peripheral blood T cells with antigen-expressing CD14-ML-DC generated by this procedure successfully induced CD8^+^ T cell lines that were reactive to CMVpp65 or MART1/Melan-A.

Anti-cancer vaccination therapies with antigenic peptides are currently conducted at many institutions. The synthetic peptide-based vaccination therapy requires information on the sequence of the epitopes for the cancer antigens. One of the drawbacks of the peptide vaccine is that so far, the identified epitopes are limited to those restricted by common HLA class I alleles, such as HLA-A*02:01 [1–4]. Thus, a considerable number of patients that are negative for major alleles are currently excluded from vaccination therapies. Another problem may be that HLA-B-restricted epitopes have been hard to identify.

The generation of antigen-expressing CD14-ML-DC should be feasible from any donor. Efficient T cell stimulation and the induction of antigen-reactive T cell lines demonstrated in the current study, imply that CD14-ML-DC can efficiently work in vaccination therapy. We consider that it is possible that the technology based on the CD14-ML will be able to change the current situation of anti-cancer vaccination therapy. Determination of cancer antigen epitopes may become unnecessary, and all cancer patients, irrespective of HLA type, will benefit from vaccination therapy.

## Supporting Information

S1 Fig Morphology of monocytes introduced with cMYC, BMI1, plus various factors. Photo images of CD14^+^ monocytes at 2, 3, and 4 weeks after introduction of expression vectors for cMYC, BMI1, plus various factors are shown (**A-G**). The CD14^+^ monocyte sample used in this experiments were not able to proliferate by introduction of cMYC plus BMI1 only. In all conditions, the cultures started with 5×10^5^ monocytes and continued in the presence of M-CSF and GM-CSF.
(PPTX)

S2 Fig Comparison of proliferation of monocytes treated by old and new methods. Monocytes were introduced with cMYC and BMI1 (old method; squares), or cMYC, BMI1 and BCL2 (new method; circles) and cultured in the presence of M-CSF and GM-CSF. The culture started with 5×10^5^ monocytes in a well of 24-well culture plates. **A** and **B** show the results of experiments with monocyte samples derived from 2 different donors.
(PPTX)

S3 Fig Response of MART-1-specific CD8^+^ T cells to a melanoma cell line expressing MART-1 protein. CD8^+^ T cells were obtained from an HLA-A*02:01-positive healthy donor and co-cultured with autologous CD14-ML-DC/MART1. On day 21, the T cells were harvested and co-cultured with an HLA-A*02:01-positive MART-1-expressing melanoma cell line SK-MEL-5. Production of IFN-γ by the T cells was detected by ELISPOT assay. CD8^+^ T cells derived from the same donor and pre-stimulated with an HIV peptide (HLA-A*02:01-restricted)-loaded CD14-ML-DC were used as control T cells.
(PPTX)

S1 Table Fold increase of cell number at 6 weeks after introduction of various factors along with cMYC plus BMI1 (DOCX)
