# Supplementary material for: Generation of Large Numbers of Antigen-Expressing Human Dendritic Cells Using CD14-ML Technology
Source: PLoS One. 2016 Apr 6;11(4):e0152384. doi: 10.1371/journal.pone.0152384 (PMC4822879; doi:10.1371/journal.pone.0152384)
Supplement: S1 Fig — Photo images of CD14+ monocytes at 2, 3, and 4 weeks after introduction of expression vectors for cMYC, BMI1, plus various factors are shown (A-G). The CD14+ monocyte sample used in this experiments were not able to proliferate by introduction of cMYC plus BMI1 only. In all conditions, the cultures started with 5×10^5^ monocytes and continued in the presence of M-CSF and GM-CSF. (PPTX) [file pone.0152384.s001.pptx]

## Slide 1
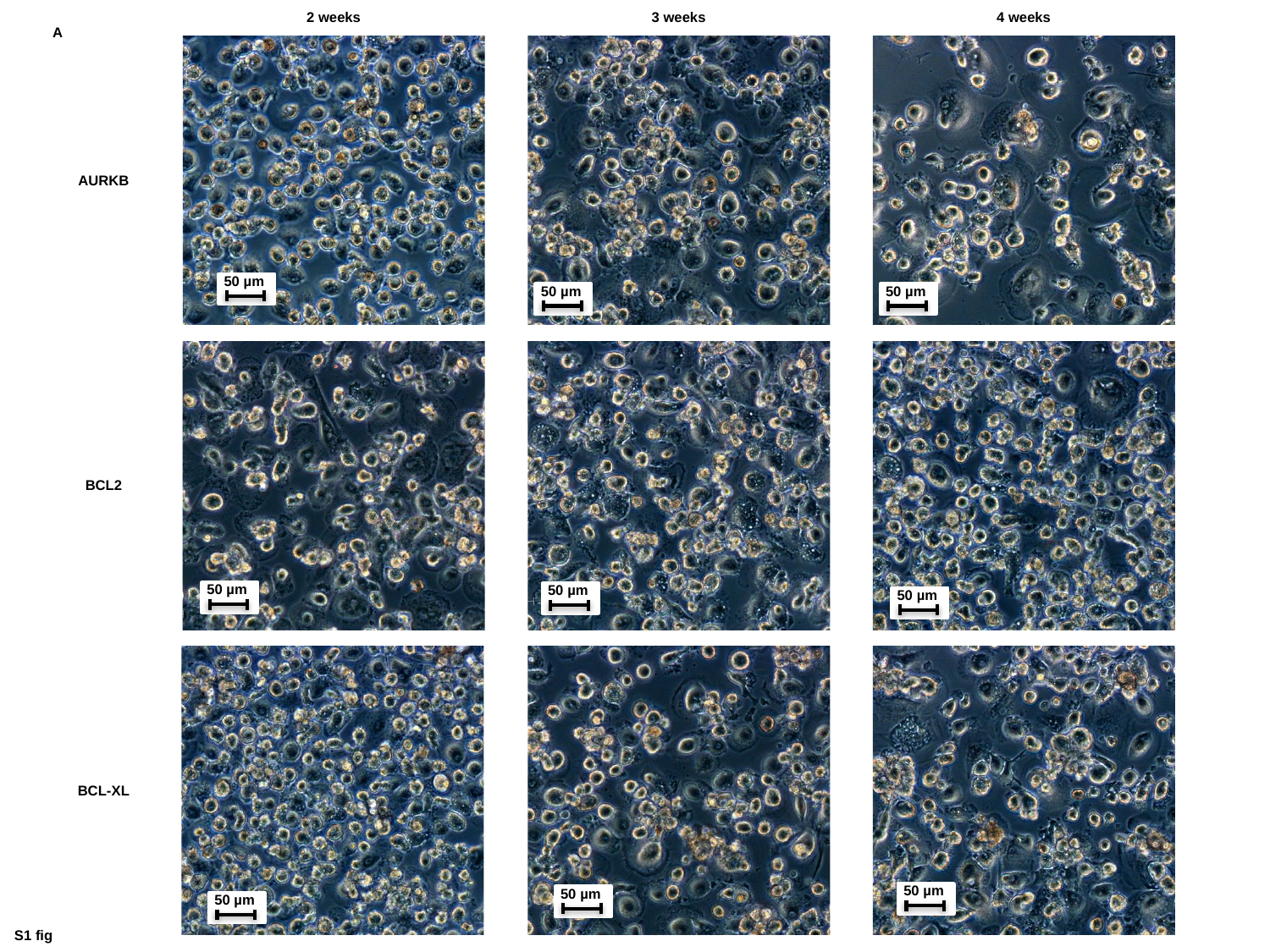

2 weeks
3 weeks
4 weeks
A
50 µm
50 µm
50 µm
AURKB
50 µm
50 µm
50 µm
BCL2
50 µm
50 µm
50 µm
BCL-XL
S1 fig

## Slide 2
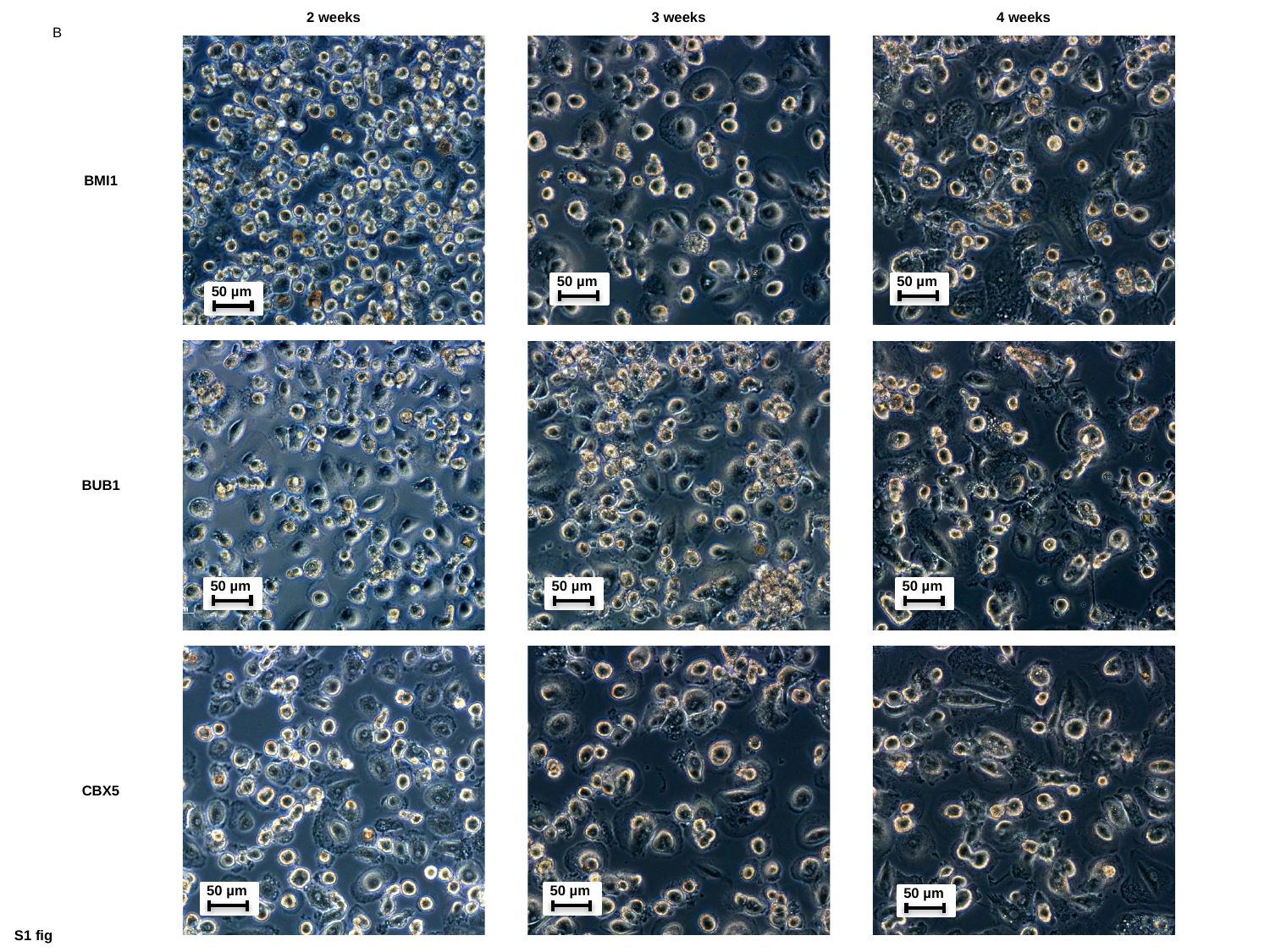

2 weeks
3 weeks
4 weeks
B
50 µm
50 µm
50 µm
BMI1
50 µm
50 µm
50 µm
BUB1
50 µm
50 µm
50 µm
CBX5
S1 fig

## Slide 3
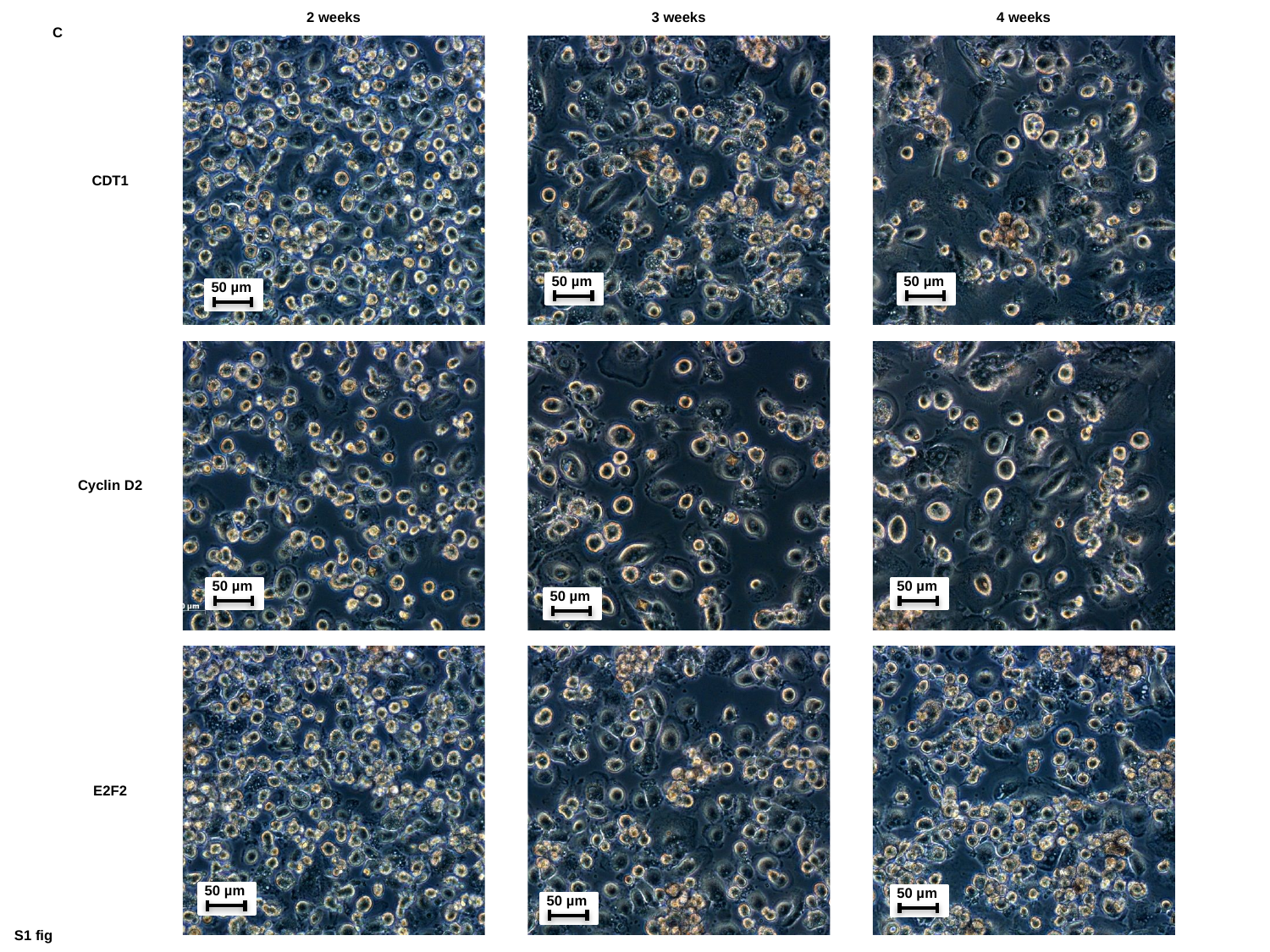

2 weeks
3 weeks
4 weeks
C
50 µm
50 µm
50 µm
CDT1
50 µm
50 µm
50 µm
Cyclin D2
50 µm
50 µm
50 µm
E2F2
S1 fig

## Slide 4
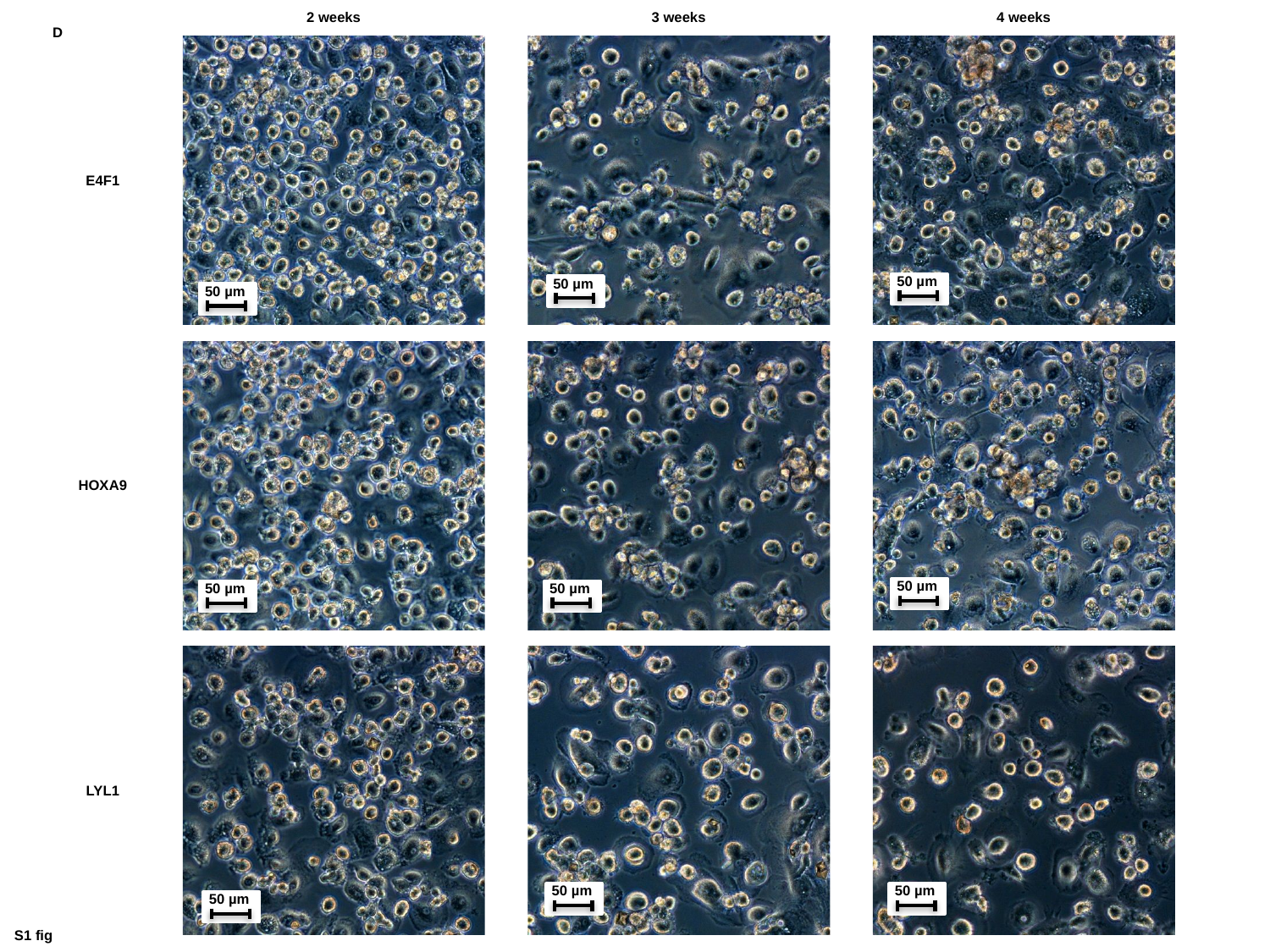

2 weeks
3 weeks
4 weeks
D
50 µm
50 µm
50 µm
E4F1
50 µm
50 µm
50 µm
HOXA9
50 µm
50 µm
50 µm
LYL1
S1 fig

## Slide 5
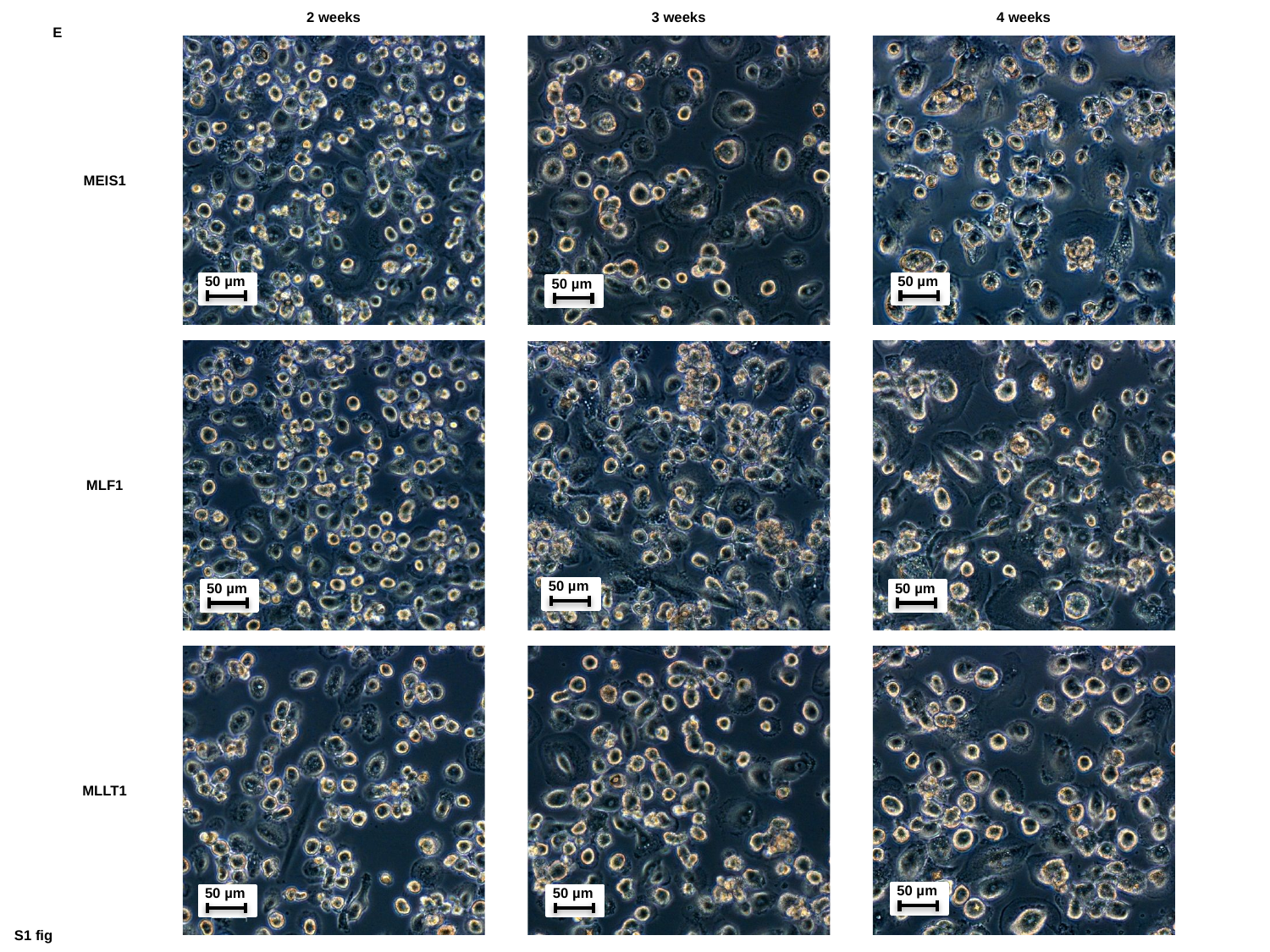

2 weeks
3 weeks
4 weeks
E
50 µm
50 µm
50 µm
MEIS1
50 µm
50 µm
50 µm
MLF1
50 µm
50 µm
50 µm
MLLT1
S1 fig

## Slide 6
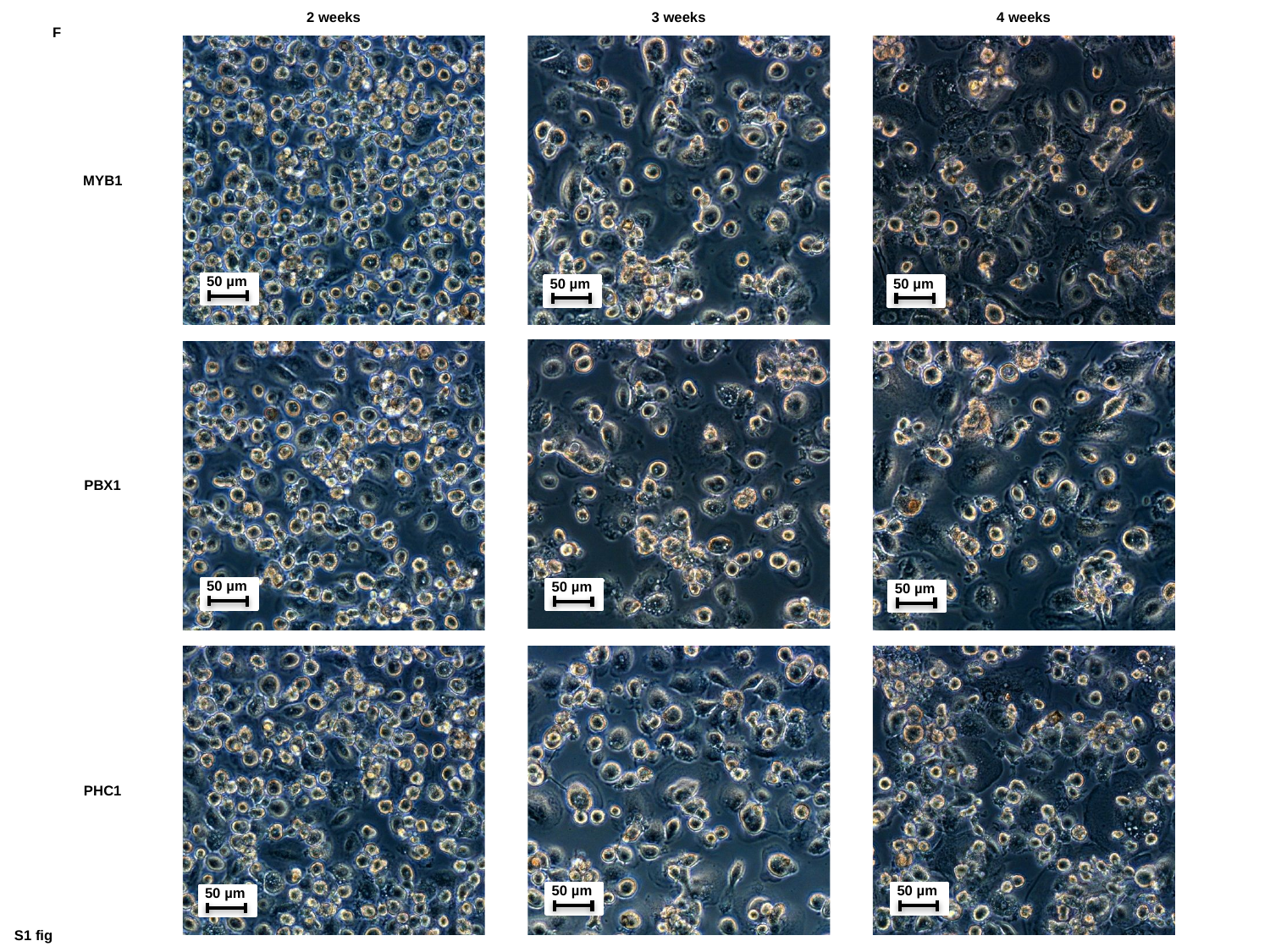

2 weeks
3 weeks
4 weeks
F
50 µm
50 µm
50 µm
MYB1
50 µm
50 µm
50 µm
PBX1
50 µm
50 µm
50 µm
PHC1
S1 fig

## Slide 7
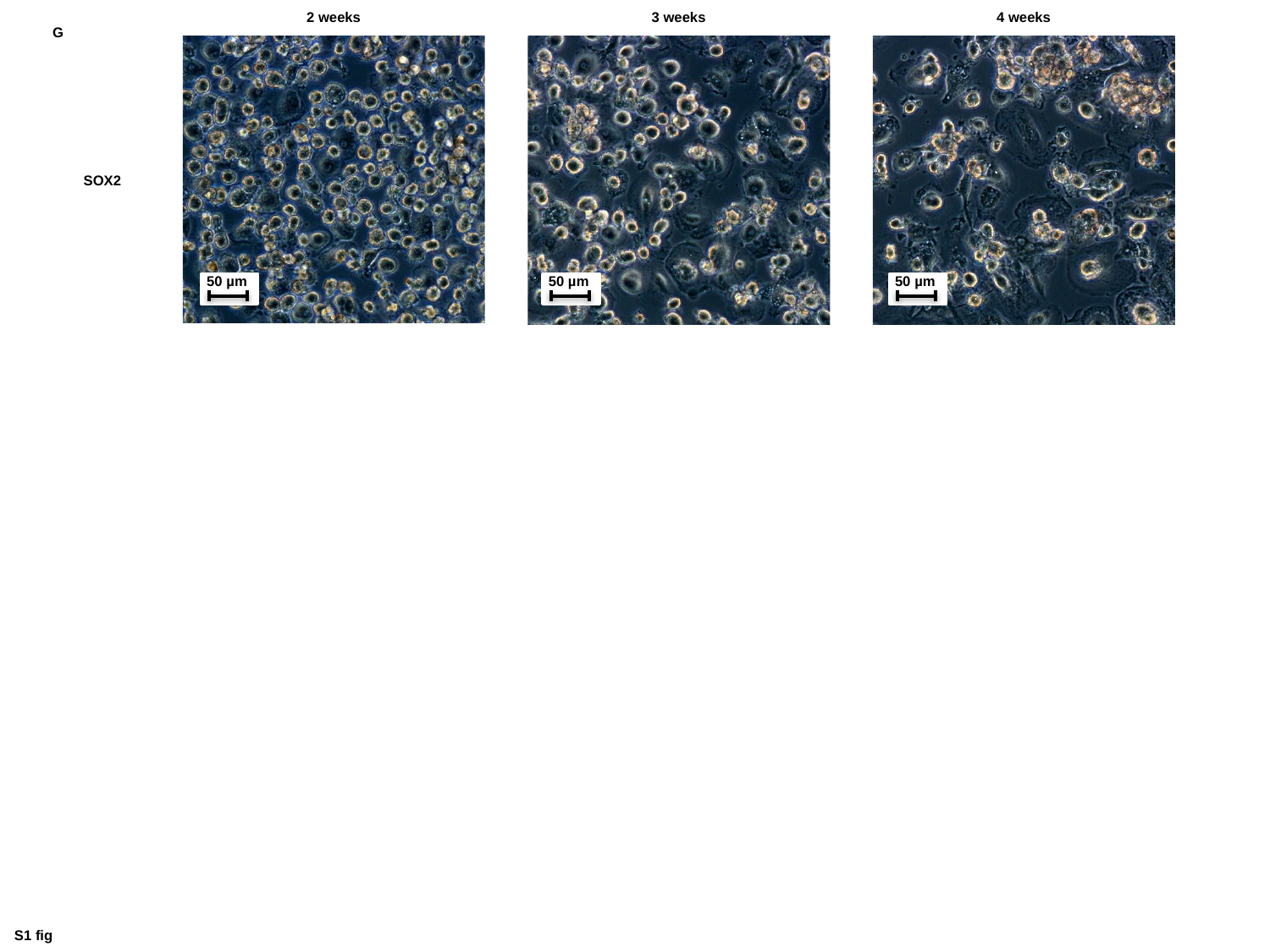

2 weeks
3 weeks
4 weeks
G
50 µm
50 µm
50 µm
SOX2
S1 fig
