# Supplementary figures and images for: Generation of Large Numbers of Antigen-Expressing Human Dendritic Cells Using CD14-ML Technology
Source: PLoS One. 2016 Apr 6;11(4):e0152384. doi: 10.1371/journal.pone.0152384 (PMC4822879; doi:10.1371/journal.pone.0152384)

## Slide 1
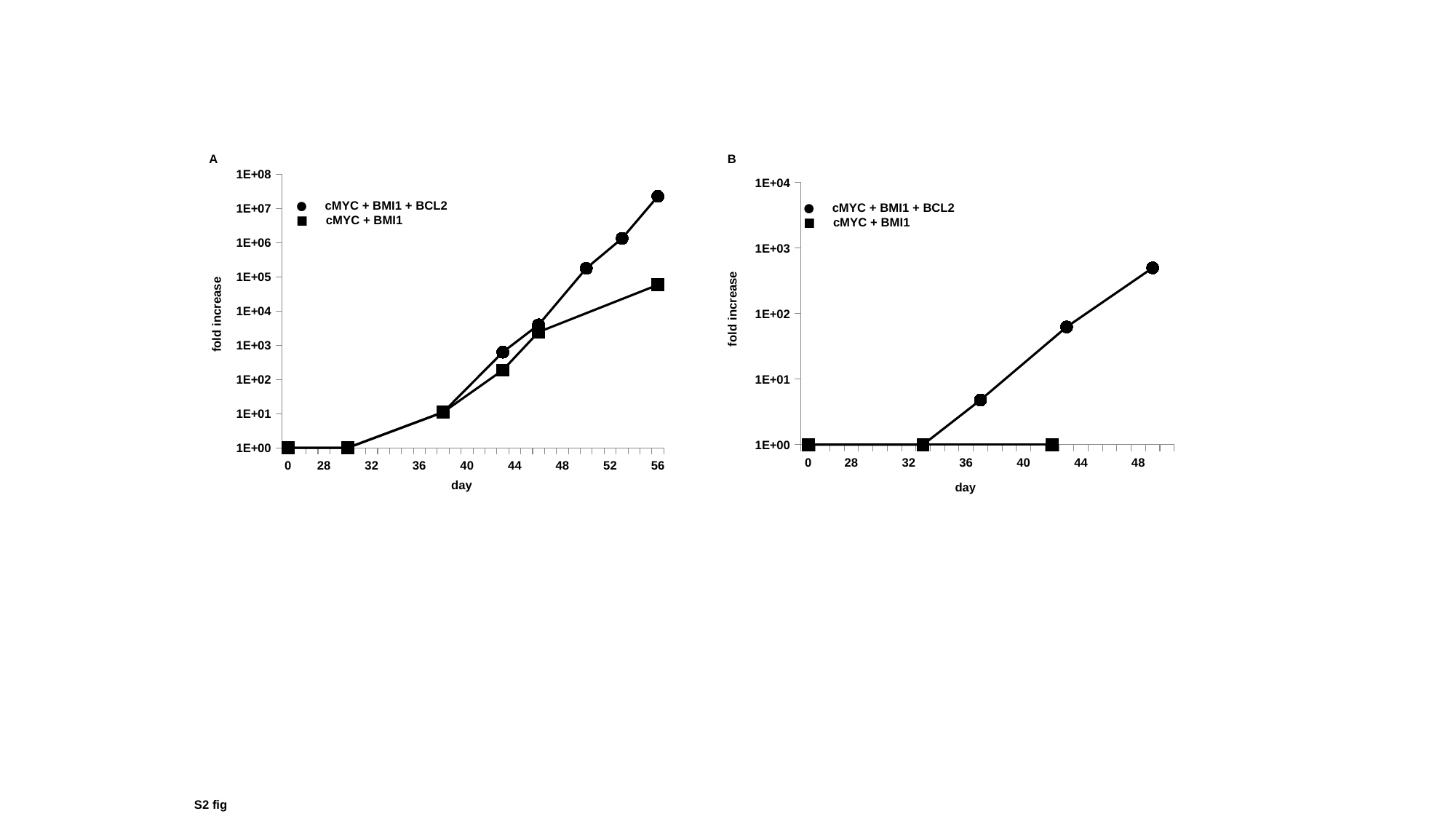

Supplement: S2 Fig — Monocytes were introduced with cMYC and BMI1 (old method; squares), or cMYC, BMI1 and BCL2 (new method; circles) and cultured in the presence of M-CSF and GM-CSF. The culture started with 5×10^5^ monocytes in a well of 24-well culture plates. A and B show the results of experiments with monocyte samples derived from 2 different donors. (PPTX) [file pone.0152384.s002.pptx]

## Slide 1
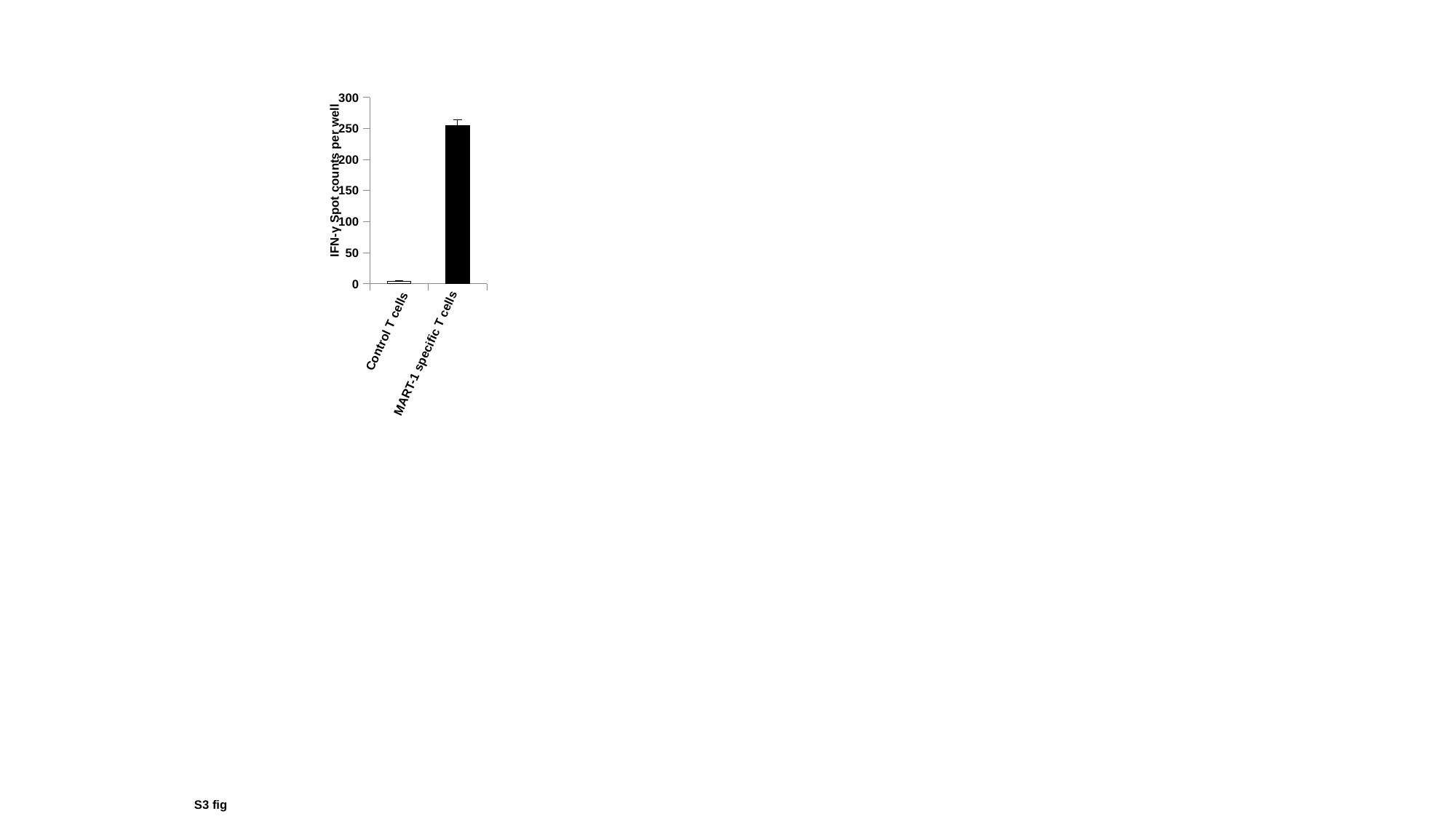

### Chart
| Category | |
|---|---|IFN-γ Spot counts per well
Control T cells
MART-1 specific T cells
S3 fig

Supplement: S3 Fig — CD8+ T cells were obtained from an HLA-A*02:01-positive healthy donor and co-cultured with autologous CD14-ML-DC/MART1. On day 21, the T cells were harvested and co-cultured with an HLA-A*02:01-positive MART-1-expressing melanoma cell line SK-MEL-5. Production of IFN-γ by the T cells was detected by ELISPOT assay. CD8+ T cells derived from the same donor and pre-stimulated with an HIV peptide (HLA-A*02:01-restricted)-loaded CD14-ML-DC were used as control T cells. (PPTX) [file pone.0152384.s003.pptx]
