# Supplementary material for: Generation of Large Numbers of Antigen-Expressing Human Dendritic Cells Using CD14-ML Technology
Source: PLoS One. 2016 Apr 6;11(4):e0152384. doi: 10.1371/journal.pone.0152384 (PMC4822879; doi:10.1371/journal.pone.0152384)
Supplement: S1 Table — (DOCX) [file pone.0152384.s004.docx]

**S1 Table. Fold increase of cell number at 6 weeks after introduction of various factors along with cMYC plus BMI1**

| Factor introduced along with cMYC plus BMI1 | Fold change of cell number |
| --- | --- |
| None | < 0.2 |
| AURKB | < 0.2 |
| BCL2 | 10.0 |
| BCL-XL | < 0.2 |
| BUB1 | < 0.2 |
| CBX5 | < 0.2 |
| CDT1 | < 0.2 |
| Cyclin D2 | < 0.2 |
| E2F2 | < 0.2 |
| E4F1 | < 0.2 |
| HOXA9 | < 0.2 |
| LYL1 | 14.6 |
| MEIS1 | < 0.2 |
| MLF1 | < 0.2 |
| MLLT1 | < 0.2 |
| MYB1 | < 0.2 |
| PBX1 | < 0.2 |
| PHC1 | < 0.2 |
| SOX2 | < 0.2 |
